# Effect of Teacher Support on Adolescents’ Positive Academic Emotion in China: Mediating Role of Psychological Suzhi and General Self-Efficacy

**DOI:** 10.3390/ijerph192416635

**Published:** 2022-12-11

**Authors:** Xu Chen, Hongxia Zhao, Dajun Zhang

**Affiliations:** 1 Normal College, Jimei University, Xiamen 361021, China; 2 Psychological Department, Southwest University, Chongqing 400715, China

**Keywords:** teacher support, psychological *suzhi*, general self-efficacy, positive academic emotion, adolescents

## Abstract

Positive academic emotions can promote good academic performance and development in students. Therefore, how teachers stimulate students to produce more positive academic emotions is particularly important. This study aimed to reveal the underlying mechanism of teacher support and adolescents’ positive academic emotions. A total of 854 students from western China participated in this survey, which included the Students’ Perception of the Teacher’s Behavioural Support Questionnaire, the Psychological Suzhi Questionnaire for Middle School Students, the General Self-Efficacy Scale, and the Achievement Emotions Questionnaire (396 boys; 12 to 20 years old, M_age_ = 15.3, SD = 2.04). Results showed that (1) teacher support, psychological *suzhi*, and general self-efficacy were positively correlated with students’ positive academic emotion; (2) psychological *suzhi* and general self-efficacy played a separate mediating role between teacher support and adolescents’ positive academic emotion; and (3) teacher support also influenced adolescents’ positive academic emotion through the serial mediation of psychological *suzhi* and general self-efficacy.

## 1. Introduction

Adolescents experience positive academic emotions, such as enjoyment, hope, and pride, in school settings, which contribute to their development [1,2]. Numerous studies have indicated that positive academic emotions could increase adolescents’ achievement motivation and academic achievement [3,4,5,6]. Other studies also found that positive academic emotions could effectively predict positive mental characteristics of adolescents, such as greater well-being and lower depression status [7,8]. Although positive academic emotion has attracted considerable attention from researchers, it is not yet clear how external teacher-related factors, such as teacher support, influence positive academic emotion of students. Understanding the influence of teachers on students’ emotional experience is crucial to improve teachers’ teaching ability and students’ academic performance and to promote healthy psychological growth of students. Therefore, the current study aimed to reveal the underlying mechanism between teacher support and adolescents’ positive academic emotion and the roles of psychological *suzhi* and general self-efficacy in this mechanism.

### 1.1. Teacher Support and Positive Academic Emotion

Teachers are important people for adolescents at school, and their behaviors have an important influence on adolescents’ academic achievement and mental health [9,10]. As a positive factor in the teacher–student interaction, previous studies have confirmed that teacher support not only predicts adolescents’ smartphone addiction and mental well-being [11,12], but also influences their career ambivalence and adaptability [13]. Indirect research has shown that teacher support helps students create a favorable class atmosphere and establish an amicable teacher–student relationship [14,15]. In this atmosphere and with this relationship, students may be relaxed and experience more positive emotions. Direct research has found that teacher support can positively facilitate adolescents’ academic emotions [16]. When adolescents perceive this supportive behavior or emotion provided by their teacher, they are more likely to feel enjoyment, hope, and pride in academic settings [15].

According to the cognitive theory of emotion, human emotional activities arise from the cognitive process and the continuous evaluation of the relationship between stimulus situations and the individual [17]. Although teacher support is an important factor that can predict adolescents’ positive academic emotion, this external factor usually requires some internal factors to take effect, including the students’ cognitive and evaluation processes [15,18].

### 1.2. The Cognitive Process: Psychological Suzhi

In recent years, with the deepening of research on psychological *suzhi*, researchers have begun to pay more attention to the influence of psychological *suzhi* on individual psychological development, especially people’s emotions [15]. An indigenous Chinese concept, psychological *suzhi* is an inherent and stable mental or psychological quality and has been described in the Handbook of Positive Psychology in Schools (Second Edition). Psychological *suzhi* is divided into three dimensions: cognitive quality, individuality, and adaptability [19,20]. Cognitive quality, the most basic component, is manifested through individuals’ cognitive processing of the “object”. Individuality is reflected through individuals’ actions towards the object. Adaptability refers to the ability of individuals to achieve consonance between themselves and the environment during socialization [20].

Studies have shown that psychological *suzhi* may boost adolescents’ mental health [21,22]. Adolescents with a high level of psychological *suzhi* are less likely to be depressed or anxious in social communication [15,23]. More importantly, psychological *suzhi* may significantly predict adolescents’ positive emotion and emotion regulation [24,25]. The higher the level of psychological *suzhi*, the more positive emotions are experienced and the more positive emotion regulation strategies are adopted. Therefore, we infer that psychological *suzhi* could increase adolescents’ experiences of positive emotion in academic settings.

Bronfenbrenner (1983) noted that the school environment, including teacher factors, was the most influential microsystem (in addition to the family environment) for children and adolescents’ psychological development. As an individual internal factor, psychological *suzhi* is influenced by microsystems. Thus, we infer that teacher support could boost adolescents’ psychological *suzhi*. Therefore, we suggest that psychological *suzhi* plays a mediating role in the relationship between teacher support and positive academic emotion.

### 1.3. The Evaluation Process: General Self-Efficacy

General self-efficacy is defined as the subjective assessment of how people could use their skills to complete a job or activity [26,27,28]. People with high self-efficacy believe that they have the ability to complete certain tasks and have a high sense of control over these tasks [29]. As suggested by Pekrun’s control value theory of academic emotions, adolescents’ emotions in academic settings come from a sense of control in relation to learning activities [30]. Therefore, general self-efficacy might have consequences for adolescents’ academic emotions, especially positive academic emotion. Some evidence has also been provided by empirical research. Sorić (2006) found that general self-efficacy had a close relationship with academic emotions; the higher the level of perceived general self-efficacy was, the more positive emotion was experienced [31]. Wang’s study (2010) confirmed that general self-efficacy significantly influenced the positive academic emotion of high school students [32]. Thus, self-efficacy was a notable internal predictor of understanding the adolescents’ positive academic emotion. General self-efficacy was also impacted by school factors, especially the relationship between teachers and their students [33]. Teacher support might influence adolescents’ general self-efficacy to some extent. Based on these findings, we infer that general self-efficacy is a mediator in the relationship between teacher support and positive academic emotion.

### 1.4. Psychological Suzhi and General Self-Efficacy

Psychological *suzhi*, which contains aspects of cognitive quality, individuality, and adaptability [20], has been shown to significantly predict self-esteem [34]. Bandura (1989) showed that the cognitive process is one of the most important influencing factors for general self-efficacy [27]. Some studies have indicated certain personality factors that influence general self-efficacy [35,36,37]. For example, high conscientiousness, extraversion, and openness to experience reveal a tendency to high scores related to the sense of self-efficacy, and the personality trait of conscientiousness plays a greater role than other personality traits in another study [36,37]. Furthermore, general self-efficacy can decrease in a greater variety of tasks for people with low self-esteem than for those with high self-esteem [35], and adolescents’ self-esteem may predict their academic general self-efficacy [38]. Accordingly, we infer that psychological *suzhi* could impact adolescents’ general self-efficacy.

### 1.5. The Current Study

The current study aims to examine the underlying mechanism of the influence of teacher support on adolescents’ positive academic emotion and to reveal the specific effects of psychological *suzhi* and general self-efficacy on this relationship. On the basis of the literature review, we developed the following three research hypotheses.

**Hypothesis** **1** **(H1).** *Teacher support, psychological suzhi, and general self-efficacy are positively related to positive academic emotion.*

**Hypothesis** **2a** **(H2a).** *Psychological suzhi mediates the relationship between teacher support and positive academic emotion.*

**Hypothesis** **2b** **(H2b).** *General self-efficacy mediates the relationship between teacher support and positive academic emotion.*

**Hypothesis** **3** **(H3).** *Teacher support has multiple mediating effects on positive academic emotion by psychological suzhi via general self-efficacy.*

## 2. Methods

In this study, the questionnaire method was mainly used to collect research data. Measuring tools included the Students’ Perception of Teacher’s Behavioural Support Questionnaire, the Psychological Suzhi Questionnaire for Middle School Students, the General Self-Efficacy Scale, and the Academic Emotion Questionnaire for Adolescents. In addition, the participants in the study were recruited mainly by voluntary means in middle schools. Details are described below.

### 2.1. Participants and Process

Data were collected from a sample of 854 Chinese adolescents, including 396 male (46.3%) and 458 female (53.7%). Participants were selected from the 7th to 12th grade of middle schools and high schools, with 98 adolescents of the 7th grade, 189 of the 8th grade, 115 of the 9th grade, 169 of the 10th grade, 186 of the 11th grade, and 161 of the 12th grade. The average age of these participants was 15.30 years (*SD* = 2.04), with a range of 12 to 20 years.

In this study, participants were recruited according to the principle of voluntariness and convenient sampling. In the area where the researchers are located, after asking for the permission and informed consent of the principals and head teachers of qualified middle schools, a cluster sampling was conducted for students in grades 7 to 12 in a class. Only students who knew the purpose of this survey and were willing to participate in the survey (with informed consent) could be measured. All participants were required to complete four self-reported questionnaires in 40 min under the guidance of trained researchers, and the completed questionnaires were then collected by the researchers.

### 2.2. Measures

#### 2.2.1. Teacher Support

Teacher support was measured with a published Chinese version of the Students’ Perception of Teacher’s Behavioural Support Questionnaire (SPTSQ) [39]. This scale consists of 19 items with responses on a six-point Likert-type scale (from “No, totally inconsistent” to “Yes, totally consistent”). It includes three dimensions: learning support, teacher emotional support, and ability support. A sample item is “In my studies, the teacher is very strict with me”. The SPTSQ has been demonstrated to be a reliable and valid measurement [39]. In this study, the scale had good internal consistency. The internal consistency reliability coefficients (Cronbach’s *α*) of the total scale and three subscales (learning support, emotional support, and ability support) were 0.864, 0.702, 0.759, and 0.712.

#### 2.2.2. Psychological *Suzhi*

Psychological *suzhi* was examined using the Psychological *suzhi* Questionnaire for Middle School Students (PSMQ) [23], which includes 24 items and 3 dimensions (cognitive quality, individuality, and adaptability). Each dimension has 8 items. Sample items are as follows: “I am good at linking old and new knowledge to my studies”; “I usually do my own thing by myself”; and “I often can resolve embarrassment effectively and respectfully”. Responses to all items were on a five-point Likert-type scale (from “Totally disagree” to “Totally agree”). The PSMQ is suitable for adolescents in the Chinese school environment [15], and has good internal consistency and reliability for the total scale [23]. In this study, Cronbach’s *α* of the scale was 0.855.

#### 2.2.3. General Self-Efficacy

We adopted the Chinese version of the General Self-Efficacy Scale (GSES) to examine the general self-efficacy of Chinese adolescents [40]. The scale included 10 items (a sample item is “If I try my best to do it, I can always solve the problem”), which were answered on a four-point Likert-type scale (from “Totally wrong” to “Totally right”). Previous studies have found that the Chinese version of the GSES is a reliable and valid measurement [40,41]. In this study, the scale had good internal consistency (Cronbach’s *α* = 0.813).

#### 2.2.4. Positive Academic Emotion

According to the Achievement Emotions Questionnaire, Dong and Yu (2007) designed the Academic Emotion Questionnaire for Adolescents (AEQA) [2]. The AEQA has 72 items. However, in the current study, we adopted the 30 items that belong to positive academic emotion (a sample item is “I think learning is fun”). All items from this scale were answered on a five-point Likert-type scale (from “Totally disagree” to “Totally agree”). This questionnaire has been shown to have good reliability and validity [42]. In this study, the Cronbach’s *α* of positive academic emotion was 0.798.

### 2.3. Data Analysis

First, Harman’s single factor analysis was used to test for possible bias using the common method in the current study. Then, we performed descriptive and correlative analyses of the main variables. The mediating effects of psychological *suzhi* and general self-efficacy were then examined by structural equation analysis and bias-corrected bootstrap. Finally, we examined the multiple mediating effects of psychological *suzhi* and self-efficacy on the relationship between teacher support and positive academic emotion.

The above analysis was conducted with SPSS21.0 and Amos21.0. Regarding the existing recommendations on the fitting indexes of the structural equation model (SEM), we adopted many common indexes to evaluate the model in the current study, including the chi-square value (χ^2^), degrees of freedom (*df*), ratio of the chi-square to degrees of freedom (χ^2^/*df*), root mean square residual (RMR), root mean square error of approximation (RMSEA), goodness-of-fit index (GFI), normed fit index (NFI), incremental fit index (IFI), Tucker–Lewis index (TLI), and comparative fit index (CFI). If a model is acceptable, the χ^2^ should be non-significant, the χ^2^/*df* should be less than 5, and other indexes should be greater than 0.90 [43,44,45]. We adopted the abbreviated forms of the variables of interest in the results (TS = teacher support; P*s* = psychological *suzhi*; GSE = general self-efficacy; PAE = positive academic emotion).

## 3. Results

### 3.1. Common Method Bias Test

Due to the use of the four self-report questionnaires that the participants completed in the same place, there might be a bias of the common method in the current study. Thus, it is necessary to test whether this bias is significant. Harman’s single-factor analysis was used to test for this potential bias. Results showed that a total of 20 principal components (more than one principal component) were found in this research, and the contribution rate of the first principal component was only 13.66% (less than the critical value of 40%). Accordingly, we can conclude that common method bias did not exist in the current study, and the data collected were valid.

### 3.2. Descriptive Statistics of Variables

Table 1 reports the descriptive and correlative analysis of all variables of interest, including means, standard deviations, and correlations. The results indicated that TS, PS, and GSE were significantly and positively correlated with PAE (*r* = 0.25 to 0.54, *p* < 0.01). Additionally, there were significant positive relationships of TS and PS with GSE (*r* = 0.25 to 0.54, *p* < 0.01). TS was also significantly positively associated with PS (*r* = 0.25 to 0.54, *p* < 0.001).

### 3.3. The Mediating Effects of Psychological Suzhi and General Self-Efficacy

**Figure 1:**
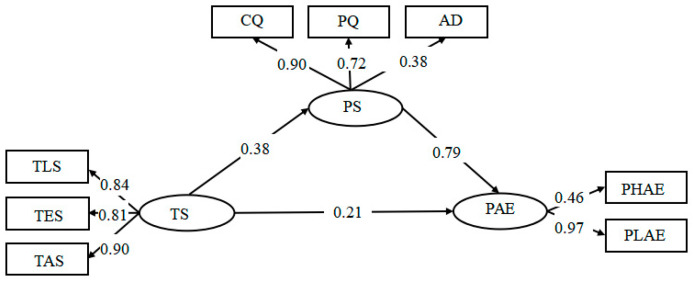
SEM with TS, PS, and PAE. TLS = teacher learning support, TES = teacher emotional support, TCS = teacher ability; CQ = cognitive quality; PQ = personality quality; AD = adaptability; PHAE = positive high-arousal academic emotion; PLAE = positive low-arousal academic emotion.

We first tested the mediating model among PS, TS, and PAE. The results of the SEM analysis showed that this model was acceptable, χ^2^ = 59.67, *p* < 0.001, *df* = 15, χ^2^/*df* = 3.98, RMR = 0.03, RMSEA = 0.06 (90% CI: 0.10, 0.15), GFI = 0.98, NFI = 0.97, IFI = 0.98, TLI = 0.96, CFI = 0.98. The path coefficients of TS to PS and from TS and PS to PAE were significant (*β* = 0.375 to 0.21, *p* < 0.001). Therefore, PS had a mediating effect on the relationship between TS and PAE, and the results support H2a. The detailed results of this model are shown in Figure 1.

**Figure 2:**
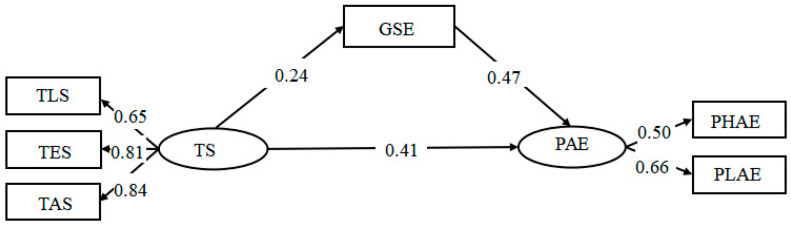
SEM with TS, GSE, and PAE.

Next, we examined the mediating effect of GSE. The results showed that the mediating model had an acceptable fit to the data, χ^2^ = 28.37, *p* < 0.001, *df* = 6, χ^2^/*df* = 4.73, RMR = 0.03, RMSEA = 0.07 (90% CI: 0.01, 0.05), GFI = 0.99, NFI = 0.98, IFI = 0.98, TLI = 0.96, CFI = 0.98. TS had a significant direct effect on adolescents’ PAE (*β* = 0.41, *p* < 0.001) and a significant indirect effect through GSE (*β* = 0.24 to 0.47, *p* < 0.001); these results supported H2b. For detailed results, see Figure 2.

### 3.4. The Multiple Mediating Effects between Teacher Support and Positive Academic Emotion

**Figure 3:**
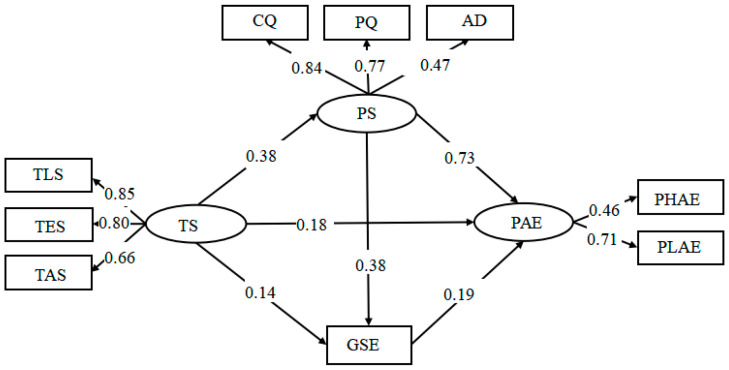
SEM with TS, PS, GSE, and PAE.

We also tested the combined effects of PS and GSE in the relationship between TS and PAE. The SEM results indicated that the fit of the comprehensive model was acceptable, χ^2^ = 78.30, *p* < 0.001, *df* = 19, χ^2^/*df* = 4.12, RMR = 0.03, RMSEA = 0.06 (90% CI: 0.05, 0.08), GFI = 0.98, NFI = 0.97, IFI = 0.98, TLI = 0.96, CFI = 0.98. The SEM results showed that the indirect path from TS to PAE was significant (*β* = 0.18, *p* < 0.001). Furthermore, the indirect paths from TS and PS to PAE (*β* = 0.38 to 0.73, *p* < 0.001) and TS and GSE to PAE were significant (*β* = 0.14 to 0.19, *p* < 0.001). Most importantly, the multiple mediating paths of chain type from TS, PS, and GSE to PAE were significant (*β* = 0.19 to 0.73, *p* < 0.001); these results verified H3 in this study. For detailed results, see Figure 3.

## 4. Discussion

The current study examined the underlying mechanism of the influence of teacher support on middle school adolescents’ positive academic emotion and revealed the specific effects of psychological *suzhi* and general self-efficacy. Our findings indicated that teacher support, psychological *sushi*, and general self-efficacy had a significant correlation with positive academic emotion. In addition, psychological *suzhi* and general self-efficacy played a mediating role in the relationship between teacher support and positive academic emotion, and teacher support had multiple mediating effects on positive academic emotion by psychological *suzhi* through general self-efficacy. These findings support the hypotheses proposed in this study.

### 4.1. Teacher Support and Positive Academic Emotion

Consistent with previous research, teacher support was an important predictor of adolescents’ positive academic emotion [15,16]. That is, adolescents will have more positive academic emotions, such as excitement, joy, and relaxation, if they receive or perceive more help from their teacher. The reason for this finding is that teacher enthusiasm is significantly related to secondary school adolescents’ enjoyment of learning and hope for success [30,46], and through the process of emotional contagion, a teacher’s enthusiasm might stimulate excitement and positive affect in adolescents [30].

### 4.2. The Mediating Effect of Psychological Suzhi

Psychological *suzhi* significantly mediated the relationship of teacher support with positive academic emotion, which is in agreement with Lazarus’s cognitive theory of emotion and Bronfenbrenner’s theory of ecological systems of human development [17,47]. A possible explanation is that the teacher as a kind of social support, who has a general stimulating effect on individual internal psychology and mental development [48,49], and psychological *suzhi* is an important part of individual-oriented positive psychology [19], which could significantly increase positive emotion [50]. Adolescents who receive more support from teachers are likely to develop higher levels of psychological *suzhi.* Due to the support provided by psychological *suzhi,* these adolescents experience more positive emotions in the academic environment.

### 4.3. The Mediating Effect of General Self-Efficacy 

The findings also indicated that general self-efficacy was a positive mediator between teacher support and positive academic emotion. Support from the teacher may increase the confidence of adolescents, and improve the assessment of themselves in relation to academics or life [15]. This improved confidence and self-assessment may enable them to feel a sense of control or meaning related to their current activities and to experience more positive emotions [51,52], which is consistent with Pekrun’s control value theory [30].

In addition, teacher support had multiple mediating effects on positive academic emotion by psychological *suzhi* through general self-efficacy. This finding is in agreement with Bandura’s theory of self-efficacy and shows that psychological *suzhi* may effectively improve adolescents’ self-efficacy [26,27]. Teachers’ support increased adolescents’ general self-efficacy by improving their psychological *suzhi* and helped them to feel more positive emotions from their learning with a high level of general self-efficacy.

### 4.4. The Multiple Mediating Effects between Teacher Support and Positive Academic Emotion

To some extent, the findings of this study enrich theoretical research on the underlying mechanism of the influence of teacher support on students’ positive academic emotion. In the specific practice of education, these findings can enlighten educators with the idea that teachers’ behaviors profoundly influence students’ emotions during their studies and that teachers should pay more attention to adolescents in terms of support. In actual educational situations, educators should pay more attention to the psychological *sushi* and general self-efficacy of adolescents and take appropriate measures to cultivate and improve them.

## 5. Conclusions

Teacher support, psychological *sushi*, and general self-efficacy are positively correlated with positive academic emotion. Psychological *suzhi* mediates general self-efficacy and the relationship between teacher support and positive academic emotion. Teacher support not only directly and significantly predicts positive academic emotion but also indirectly predicts positive academic emotion through psychological *suzhi* and general self-efficacy. Adolescents with more teacher support are more likely to obtain higher psychological *suzhi*, which improves their level of general self-efficacy. This improved general self-efficacy helps them experience more positive emotion in academic settings.

This study inevitably has some limitations. The sample size was relatively small and the samples lack sufficient representativeness. In addition to teacher factors, many other school and family factors must be considered in relation to the formative mechanism of positive academic emotion. These results are based on a cross-sectional study and should be added to a longitudinal study in further research.

## Figures and Tables

**Table 1:** Means, SDs, correlations, and Cronbach’s alphas among TS, PS, GSE, and PAE (N = 854).

Variables	*M*	*SD*	1	2	3	4	Cronbach’s *α*
1 TS	67.27	13.81	1				0.864
2 PS	81.90	10.78	0.36 **	1			0.855
3 GSE	2.40	0.50	0.25 **	0.41 **	1		0.813
4 PAE	101.45	12.30	0.36 **	0.55 **	0.39 **	1	0.798

Note. TS = Teacher support, PS = Psychological *suzhi*, GSE = General self-efficacy, PAE = Positive academic emotion; ** *p* < 0.001.

## Data Availability

Due to the privacy of the participants, the data will not be disclosed temporarily. If necessary, please contact the corresponding author (202161000135@jmu.edu.cn) or the first author (chenxu_swu@163.com).
